# Body condition scoring in alpacas (*Vicugna pacos*) and llamas (*Lama glama*) – a scoping review

**DOI:** 10.1007/s11259-023-10275-y

**Published:** 2023-12-22

**Authors:** Matthias Gerhard Wagener, Martin Ganter, Sabine Leonhard-Marek

**Affiliations:** 1 Clinic for Swine, Small Ruminants, Forensic Medicine and Ambulatory Service, University of Veterinary Medicine Hannover Foundation, Hannover, Germany; 2 Library, University of Veterinary Medicine Hannover Foundation, Hannover, Germany; 3 Department of Physiology, University of Veterinary Medicine Hannover Foundation, Hannover, Germany

**Keywords:** South American camelids, Nutritional status, BCS, Emaciation, Clinical score

## Abstract

Assessment of the body condition score (BCS) is an important tool to check the nutritional status of an animal, and thus can provide important information in herd management, but also in the veterinary examination of individual animals. In dairy farming, BCS has been recognized as an important parameter for a long time already, but over the years body condition scoring has also become more important for other species. Especially in South American camelids (SACs: alpacas and llamas), whose dense coat can hide a poor nutritional status for a long time, regular assessment of the BCS is recommended by different authors and organizations. To date, there are several instructions for the assessment of the BCS in SACs, which, however, differ significantly in some points. For a consistent approach, a total of 35 instructions for the BCS in SACs were compared in a scoping review. The spine, especially the lumbar vertebrae as well as the ribs were identified as the two main body sites recommended for assessing the BCS. Furthermore, the area between the front and rear leg and the pelvis were mentioned frequently; however, these body sites were discussed controversially. The paralumbar fossa, shoulder, and udder were only mentioned sporadically. Among the various scales, the 1–5 scale was mentioned most frequently, with the optimal BCS being 3. Nonetheless, physiological fluctuation due to breed, age, sex, pregnancy, and lactation have to be considered. The BCS should be assessed each time the animals are handled, but at least monthly. In contrast to body weight, the BCS reflects the nutritional status of an animal even after a single examination. The review showed that much of the available information on the assessment of BCS in SACs is based on practical experience rather than scientific evidence. Indeed, some of the assumptions made there might still have to be verified or modified.

**Supplementary Information:**

The online version contains supplementary material available at 10.1007/s11259-023-10275-y.

## Introduction

The keeping of South American camelids [SACs; alpacas (*Vicugna pacos*) and llamas (*Lama glama*)] is becoming more and more popular in Europe (Davis et al. 1998; D'Alterio 2006; Hengrave Burri et al. 2005; Neubert et al. 2021; Wagner et al. 2022). The number of owners of llamas and alpacas has increased significantly in recent years (Neubert et al. 2021). Since SACs can compensate for disease symptoms for a long time, early detection of diseases is an important issue in the keeping of alpacas and llamas. This can be particularly problematic for owners who are new to keeping SACs, as they often lack the necessary sensitivity to assess the health status of the animals correctly. This results in many SACs presented at the veterinary clinic or undergoing a post-mortem examination being in poor nutritional condition (Wagener et al. 2021; Neubert et al. 2022). Nutritional status is an important parameter for assessing health in an animal (Roche et al. 2009). However, the assessment of the nutritional status is particularly challenging in SACs, as the animals have a dense coat unless they are shorn (Van Saun 2006a, 2009a; Fowler 2010). This can quickly lead to the nutritional state of the animal being hidden from the observer so that emaciated animals might go unrecognized for a long time. Therefore, similar to sheep, nutritional status in alpacas and llamas should not be assessed by visual examination of the animal alone, but by palpation (Australian Alpaca Association 2008; Russel 1984). A simple tool for standardized determination of the nutritional status is the assessment of a body condition score (BCS) (Bewley and Schutz 2008; Roche et al. 2009). This involves examining different body sites of an animal by visual inspection or palpation for assessing the muscle and fat coverage of specific bone points (Burkholder 2000). Usually, the BCS is expressed numerically on a specific scale, with the smallest value on the scale describing an emaciated animal and the highest value describing a severely obese animal (Burkholder 2000). The optimal score is usually in the middle of the scale, but physiological fluctuations around the median, such as occur during lactation, are also taken into account (Kenyon et al. 2014; Panne and Mansfeld 2022).

The BCS was originally developed for sheep in Tasmania in the 1960s by Jefferies (Jefferies 1961) and later modified by Russel et al. (Russel et al. 1969; Russel 1984). A similar system is also described for cattle by different authors (Roche et al. 2009; Wildman et al. 1982; Ferguson et al. 1994; Metzner et al. 1993) and body condition scoring plays an important role especially in dairy farming where it has been used for several decades (Roche et al. 2009; Bewley and Schutz 2008; Markusfeld et al. 1997; Panne and Mansfeld 2022). While the BCS in small ruminants is usually assessed by palpation of bone and muscle covering in the area of the last (13th) rib (Thompson and Meyer 1994; Kenyon et al. 2014), the BCS in cattle is assessed at eight different body sites (Edmonson et al. 1989; Panne and Mansfeld 2022). The more differentiated examination in cattle allows more sensitive grading which is expressed in 0.25 steps (Ferguson et al. 1994).

In addition to ruminants, body condition scoring is a common method to assess the nutritional status in many other farmed (Charette et al. 1996; Carroll and Huntington 1988; Henneke et al. 1983; Gerhart et al. 1996) and companion species (Laflamme 1997a, 1997b). It has also established itself in Old World and New World camelids and is seen as an important factor to assess camel welfare (Menchetti et al. 2021; Padalino and Menchetti 2021). In the case of Old World camelids, several body sites, including the hump, are included in the assessment of the BCS (Faye et al. 2001; Iglesias et al. 2020).

For SACs, the first description of a numerical BCS was made by Johnson in 1994 (Johnson 1994). To date, there are several descriptions available for assessing a BCS in SACs, where palpatory examination of the lumbar spine is usually recommended (Australian Alpaca Association 2008; Wagener and Ganter 2020; Bromage 2006; Hilton et al. 1998; Johnson 1994; Jones and Boileau 2009; Van Saun and Herdt 2014; Fowler 2010). In some sources, other body sites like the thorax behind the elbow, the paralumbar fossa, the area between the front and rear legs, or the pelvis are also included in the BCS assessment in SACs (Van Saun 2009b; Johnson 1994; Bromage 2006; Lopez 2021).

### The BCS and health status in alpacas and llamas

Body condition scoring is a helpful indicator of general health in herd management (Connolly et al. 2008). The association between BCS and health or economy of an animal is well known from other species. In cows, the BCS at parturition is correlated with milk yield, fertility, and health of the animal (Markusfeld et al. 1997; Roche et al. 2009). The effect of the BCS on the metabolism is also known in sheep (Caldeira et al. 2007a, b). So far, there are few data available on the relationship between BCS and health in SACs. Common causes of a low BCS in SACs are chronic infectious diseases, endoparasites, dental problems, qualitatively or quantitatively too poor nutrient supply, or an incorrect animal-food ratio (Van Saun 2009a, b; Baumgartner et al 2018; Frezzato et al. 2020). However, not every animal that suffers from one of these conditions necessarily has a lowered BCS. Whittle, who investigated the BCS of 82 Peruvian alpacas, found no difference between animals with and without endoparasitosis (Whittle 2015). Dental problems, which are very common in alpacas and llamas (Niehaus 2009) may also exist despite a good nutritional status. In a retrospective examination of tooth root abscesses in SACs, 45 of 58 of the examined animals (78%) revealed an optimal BCS (Niehaus and Anderson 2007). However, Proost et al. found that interproximal gum retraction is strongly associated with low BCS in alpacas (Proost et al. 2020).

The BCS is also associated with reproduction in SACs. In llamas that were fed restrictively the ovaries of the animals with poorer nutritional status had smaller dominant follicles than those of the control group (Norambuena et al. 2013). According to Vaughan et al., alpacas with a lower BCS have a significantly lower embryo transfer success rate than alpacas with a balanced BCS (Vaughan et al. 2013). Not only a low BCS has an influence, but also strong deviations from the optimal BCS in both directions can affect the fertility cycle (Van Saun 2008). However, Norambuena et al. investigated the reproductive success of female alpacas in terms of nutritional status and found no differences in the BCS between 20 pregnant and 15 non-pregnant animals (Norambuena et al. 2018). In male alpacas, there is a positive correlation between BCS and testicular length, which has an influence on sperm production (Abraham et al. 2015).

Alpacas and llamas with a lower BCS also reveal changes in the blood count and are more often anemic than animals with an adequate BCS. Storey et al. (Storey et al. 2017) described an association between low BCS and a decreased packed cell volume (PCV) in SACs. We also found significant positive correlations between BCS and PCV, haemoglobin, and the percentage of eosinophils in a retrospective evaluation of hospitalized alpacas and llamas (Wagener et al. 2021). In llamas, there was also a significant positive correlation between BCS and the percentage of lymphocytes, and a significant negative correlation between BCS and the percentage of neutrophils (Wagener et al. 2021).

The BCS should also be taken into account when interpreting other blood results of the respective animal (Van Saun 2009b). The assessment of the BCS allows the evaluation of a longer time period, while laboratory parameters such as non-esterified fatty acids or beta-hydroxybutyrate reflect rather short-term changes in energy metabolism (Van Saun 2009b). SACs generally require less protein and energy for maintenance than ruminants, but they require a higher protein content per unit of energy (Van Saun 2006b). When checking the protein metabolism in the laboratory, the parameters urea (or Blood Urea Nitrogen - BUN), protein, and albumin can be used in SACs as well as in ruminants (Van Saun 2009b). The microbial metabolism within the forestomach, however, does not seem to be affected by different BCS. Carroll et al. investigated the microbiome of C1 in alpacas and did not find any association with the BCS of the animals (Carroll et al. 2019).

Ultimately, there are also several indications that some infectious diseases are associated with a low BCS or bodyweight. Mentioned pathogens are *Haemonchus contortus* (Storey et al. 2017; Edwards et al. 2016), *Candidatus* Mycoplasma haemolamae (Crosse et al. 2012), *Eimeria macusaniensis* (Cebra et al. 2007), or *Mycobacterium bovis* (Ryan et al. 2008).

### Aim of the study

We published a practical description of a BCS for SACs in a German-language magazine for practicing veterinarians in 2020 (Wagener and Ganter 2020). During the literature search, we noticed that a large number of descriptions of a BCS for SACs were already available. However, to date, there is no overview of the body condition scoring systems in SACs that bundles this literature and highlights commonalities and differences in the English literature. In order to gain a comprehensive overview of the different scoring systems, we performed a systematic literature search in four different databases. Body condition scores for alpacas and llamas from different authors and backgrounds will be presented and compared.

## Material and Methods

### Review question

The purpose of this review was to provide an overview of the existing descriptions for assessing a BCS in alpacas and llamas in the English literature. Since different scoring systems and different scales are used in scientific publications, the aim of this study was to create a bundled overview of the available BCS in SACs and to compare them with each other in terms of origin, species, practical assessment, the scales used for assessment, and the interpretation of the results.

### Review protocol and eligibility criteria

We intended to provide a comprehensive overview of the constantly evolving literature, and therefore chose the technique of a scoping review (Grant and Booth 2009; von Elm et al. 2019).

The scoping review was performed according to the Preferred Reporting Items for Systematic reviews and Meta-Analyses extension for Scoping Reviews (PRISMA-ScR) Checklist (Tricco et al. 2018). The checklist from http://www.prisma-statement.org/Extensions/ScopingReviews was used for this purpose. The research question was based on the PICOS criteria (Population: alpacas and llamas; Intervention: none; Comparison: none; Outcome: Description of a BCS for alpacas or llamas with a numerical scale; Study design: all available reports).

Criteria for eligibility were that the source:

- a) Described a BCS in alpacas, llamas or both;
- b) Named at least one specific body site for assessing the BCS;
- c) Used a numerical score to express the BCS and specified upper and Lower limits for the BCS
- d) Was published in English.

Only sources which could be assigned to one of the following categories were included in the evaluation:

- a) Textbooks on veterinary medicine or husbandry (In the evaluation, only the most recent edition of the respective textbook was taken into account; previous editions were explained in a separate chapter [3.1.1.]).
- b) Scientific publications (from scientific journals with or without peer review as well as theses)
- c) Publications from breeding, welfare or governmental organizations
- d) Publications from veterinary services.

### Literature search

**Fig. 1:**
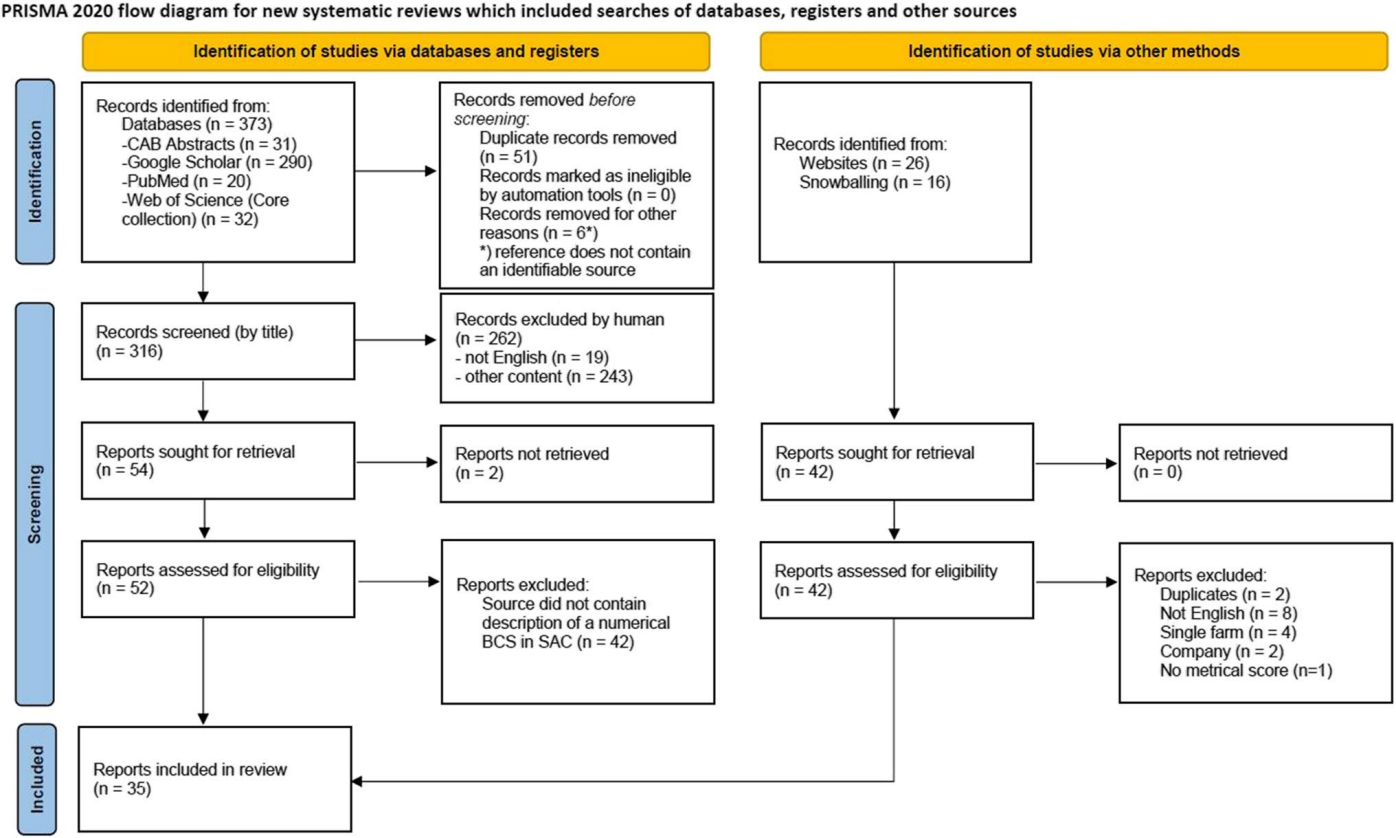
PRISMA 2020 flow diagram for the systematic literature search. From: Page MJ, McKenzie JE, Bossuyt PM, Boutron I, Hoffmann TC, Mulrow CD, et al. The PRISMA 2020 statement: an updated guideline for reporting systematic reviews. BMJ 2021;372:n71. https://doi.org/10.1136/bmj.n71. For more information, visit: http://www.prisma-statement.org/

The systematic literature search was conducted on August 29, 2022 and August 30, 2022 in four different scientific databases or platforms (Google Scholar; PubMed; Web of Science—Core Collection and CAB Abstracts [CAB Abstracts were available as two separate databases, CAB Abstracts (1910 to 1989) and CAB Abstracts (1990 onwards), the search was conducted in both databases.]). The following search term was used in each search: “((body condition score) OR (body condition scoring) OR (BCS)) AND ((alpaca) OR (vicugna pacos) OR (llama) OR (lama glama) OR (South American camelid) OR (New World camelid))”. The search results were imported into an endnote library (EndNote X9, Clarative Analytics, LLC, Philadelphia, PA, USA). Duplicates were removed automatically by the "Find Duplicates" function of endnote, which was checked manually in a second step. Records were screened according to the “PRISMA 2020 flow diagram for new systematic reviews which included searches of databases, registers and other sources”. The flow diagram (available from https://www.prisma-statement.org/PRISMAStatement/FlowDiagram) is inserted as Fig. 1.

In addition to the scientific databases, searches were also made in Google to find those body condition scoring systems, which were published by breeding, welfare, governmental associations, or veterinary services, that were not expected to be found in the scientific literature. The search terms “alpaca BCS” and “llama BCS” were used in two separate searches on August 30, 2022. In both searches, the first 100 search results were screened for matching sources. Furthermore, literature sources that were found during the research in the references of the investigated sources were added to the literature list (snowballing).

### Data analysis

The full texts of the identified sources that met the eligibility criteria for the study were read. The following information (if available) of each source was transferred to an Excel sheet (Microsoft© Excel for Office 365):

-Kind of source
-Edition (only for textbooks)
-Author(s)
-Year of publication
-Country
-Species (alpaca, llama, alpaca and llama)
-Information about the practical assessment of the BCS:
  -  Spine (lumbar region; withers)
  -  Ribs/thorax behind the elbow
  -  Shoulders
  -  Pelvis
  -  Paralumbar fossa
  -  Area between the front legs
  -  Area between the rear legs
  -  Udder (in female animals)
-Information on the accuracy of the description (detailed description of each score, indication of a specific order of examination)
-Scale used
-Optimal BCS
-Frequency of examination
-Illustrations supporting the descriptions
-Statement about body condition scoring or weighing the animals

## Results

### Types of sources

A total of 35 literature sources about body condition scoring in alpacas and llamas that met the inclusion criteria were identified. Depending on the type of source, these were assigned to one of the four groups:

- 1. Textbooks (T): *n* = 10 sources (28.6%)
- 2. Scientific publications in journals or theses (S): *n* = 11 sources (31.4%)
- 3. Breeding, welfare or governmental associations (A): *n* = 7 sources (20.0%)
- 4. Veterinary services (V): *n* = 7 sources (20.0%).

Table 1 as well as Tables S1-S4 (supplementary material) provide detailed information on the Body Condition Scoring systems in each source. To enable a faster assignment to the individual sources during reading, the numbering of the sources as given in the overview tables (T1-10; S1-11; A1-7; V1-7; see Table 1 or Tables S1-S4) is given in the continuous text. The appropriate references can be found in Table 1 and Tables S1-S4. For example, T1 is reference (Bromage 2006) and S4 is reference (Hilton et al. 1998).

**Table 1:** Overview of sources from textbooks (T1-9), scientific publications (S1-11), breeding, welfare or governmental associations (A1-7), and veterinary services (V1-7), sorted alphabetically by first author within each source type. A: alpaca; L: llama; p: the examination should be done by palpation; v: the examination should be done visually; x: it is not clear from the source whether the examination is to be performed by palpation or visually; *) the source also provides specific information about each individual score, either in writing, or as an illustration; total ranges of BCS are given in parenthesis; n.g.: optimal BCS is not given. Pelvis: Some authors specifically mentioned that the pelvis should not be included in the BCS assessment; “not” used in the table for this purpose

Source Number	Author(s)	Edition (only textbooks)	Year	Country	Species:	Scale	Optimal BCS	Illustration	Spine	Thorax behind the ellbow	Ribs	Shoulders	Pelvis	Paralumbar fossa	Area between front legs	Area between rear legs	Udder (in femals)	Reference
T1	Bromage	1	2006	UK	A;L	0–5; 1–10	3.5–4 (0–5)*	yes	p	p						v		(Bromage 2006)
T2	Duncanson	1	2012	UK	A;L	1–5; 1–10	n.g.	no	p		p	p	not					(Duncanson 2012)
T3	Fowler	2; 3	2010	USA	L	1–10	5	yes	p	p			not		p	p		(Fowler 1998); (Fowler 2010)
T4	Johnson	1	2014	USA	A;L	1–9	5	yes	p	p			not		x	x		(Johnson 2014)
T5	McConnell and Hoffman	2	2006	USA	A	1–5	3*	yes	p									(McConnell and Hoffman 2006)
T6	Niehaus ^a)^	4	2022	USA	A;L	1–5; 1–10	5 (1–10)	yes	p	p				x	x	x		(Niehaus 2022)
T7	Van Saun	2	2006	USA	A;L	1–5	3*	yes	p		p	p	p	x	v	v		(Van Saun 2006a)
T8	Van Saun^a)^	4	2022	USA	A;L	1–5	n.g.*	yes	p					x	x	x		(Van Saun 2022)
T9	Van Saun and Herdt	1	2014	USA	A;L	1–5; 1–9	3 (1–5)*	yes	p		p	p	p	x	v,p	v,p		(Van Saun and Herdt 2014)
T10	Wiedner	11	2022	USA	A	1–9	5	yes	p		p		not					(Wiedner 2022)
S1	Bennet and Richards		2015	USA	A;L	1–5; 1–9	3 (1–5)	yes	p		p				p	p		(Bennett and Richards 2015)
S2	Folkesson		2007	Sweden	A	1–5	3*	yes	p				not					(Folkesson 2007)
S3	Gomez		2011	USA	A;L	1–5	2.5–3.5*	no	p		p		p		v	v		(Gomez 2011)
S4	Hilton et al.		1998	USA	L	1–10	5	yes	p		p	p	not		v	v	v	(Hilton et al. 1998)
S5	Johnson		1994	USA	L	1–10	5	yes	p	p			not		v	v		(Johnson 1994)
S6	Jones and Boileau		2009	USA	A;L	1–10	5	yes	p	p					p	p		(Jones and Boileau 2009)
S7	Lopez		2021	USA	A;L	1–5	3*	no	p		p		p		p			(Lopez 2021)
S8	Morin et al.		1995	USA	L	1–5	n.g.	no	p		p							(Morin et al. 1995)
S9	Van Saun		2009	USA	A;L	1–5	3*	yes	p		p		p	x	v	v		(Van Saun 2009b)
S10	Van Saun		n.g	USA	A;L	1–5; 1–9	n.g.	yes	p		p		p					(Van Saun n.g.)
S11	Walker		2018	USA	A;L	1–10; (1–5)	5 (1–10)	yes	p	p	p		p		p			(Walker 2018)
A1	Alpaca Association New Zealand		2012	New Zealand	A	1–5	3*	yes	p	p					v,p	v		(Alpaca Association New Zealand 2012)
A2	Australian Alpaca Association		2008	Australia	A	1–5	2.5–3.5*	yes	p	p			not		v,p			(Australian Alpaca Association 2008)
A3	Australian Alpaca Association		n.g	Australia	A	1–5	2.5–3.5*	yes	p	p			not		x	x		(Australian Alpaca Association n.g.)
A4	British Alpaca Society (Turner)		2014	UK	A;L	1–5	3*	yes	p		p		p		v,p	v		(Turner 2014)
A5	British Alpaca Society, fact sheet		2018	UK	A	1–5	2.5–3.5*	yes	p	p			not		v,p			(British Alpaca Society 2018)
A6	Camelid Community Standards of Care Working Group		2005	USA	A;L	1–10	5–6	yes	p	p								(Camelid Community Standards of Care Working Group 2005)
A7	New Zealand Government		2018	New Zealand	A;L	1–5	3*	yes	p		p		p		v,p	v		(New Zealand Government 2018)
V1	Camelid Veterinary Services (Whitehead)		2019	UK	A	1–10	5.5–6	yes	p		p				v	v		(Whitehead 2019)
V2	Criagenesis (Vaughan)		2019	Australia	A	1–5	2.5–3	yes	p									(Vaughan 2019)
V3	Criagenesis (Vaughan)		2015	Australia	A	1–5	2.5–3*	yes	p	p			not		v,p	v,p		(Vaughan 2015)
V4	Criagenesis (Vaughan)		n.g	Australia	A	1–5	2.5*	yes	p	p			not		v,p	v,p		(Vaughan n.g.)
V5	Endell Veterinary Group (Walton and Smith)		2020	UK	A	1–5	3 *	yes	p									(Walton and Smith 2020)
V6	National Animal Disease Information Service (Potter)		2012	UK	A	1–5	2.5–3.5*	yes	p	p			not					(Potter 2012)
V7	Pennsylvania State University (Van Saun)		2013	USA	A;L	1–5	2.5–3.5*	yes	p					x	v	v		(Van Saun 2013)

**a)**: This textbook is the fourth edition of the textbook (T3). However, since the author and editor of edition 1–3 (Fowler) had died in the meantime, the textbook was revised and published by another editor (Niehaus). **b)**: The latest 11th edition was released in 2016; the website indicates that this article was last modified in 2022

#### Body condition scoring in alpacas and llamas in textbooks

Ten scoring systems (28.6%) were described in seven different textbooks, eight of which were from the USA (T3-10) and two from the UK (T1, 2). Four of the textbooks each contain one description of the BCS assessment (T1-3, 10). The other three textbooks each contain two different descriptions of a BCS in separate chapters (T4-9). In the following paragraph an insight is given into the development and the differences between the individual editions of a commonly used textbook about camelid medicine concerning the BCS in SACs.

There have been four editions of the textbook “Medicine and Surgery of Camelids” to date; the third and fourth editions have been considered separately. The author of the first three editions, M.E. Fowler, is dead so the most recent fourth edition was edited by A. Niehaus. This edition underwent a significant revision and now includes two different descriptions of the BCS (T6,8).

In the first edition of “Medicine and Surgery of Camelids”, published by Fowler in 1989, the chapter "Clinical Diagnosis: Examination and Procedures" contains the subheading "Body Condition" in the description of the physical examinations for SACs (Fowler 1989b). The author describes that palpation of the thoracic vertebrae behind the withers should be used to assess nutritional status. Palpation of the pelvis and loin is explicitly discouraged. However, classification by means of allotting a score did not yet take place in this edition. In a review paper on physical examination in llamas from the same year, the author describes the examination of body condition in a similar way (Fowler 1989a). The second and third editions of this textbook from 1998 (Fowler 1998) and 2010 (Fowler 2010) each contain the same, but further developed description of the BCS in the chapter "Feeding and Nutrition". The author recommends weighing the animals regularly, the assessment of the BCS is considered as second choice. The BCS assessment in camelids in this chapter is based on the description from Johnson (1994) (S5) by observing and palpating the withers, the fiberless areas behind the elbow, between the rear legs, the chest, and the perineum in the mentioned order. The description is supplemented by several schematic illustrations showing the individual examination sites, schematic cross-sections of the spinous processes, and the front and rear views of an animal. Photos of thin and fat llamas are also included. The ideal score is given as 5 on a scale from 1 to 10. The most recent fourth edition of “Medicine and Surgery of Camelids” published in 2022 and edited by Niehaus contains two different descriptions of a BCS, one in the chapter "Feeding and Nutrition" by van Saun (T8), the other in the chapter "Physical Exam and Diagnostics" by Niehaus (T6). Unlike Fowler's older descriptions, both newer descriptions include the paralumbar fossa in the examination, and one of the newer descriptions (T8) does not include an examination of the thorax. Further details can be found in Table 1 and Tables S1-S4.

#### Body condition scoring in alpacas and llamas in scientific publications

Eleven of the sources (31.4%) were found in scientific publications. Of these, eight (22.9%) were either review articles on nutrition (S3,5,9,10), on routine procedures in camelids (S7,11), on camelid herd health (S6), or on camelid wellness (S1) in SACs. The other three (8.6%) sources were original articles estimating the body weight of llamas (S4), the milk composition of llamas (S8), and a thesis on feeding alpacas in Sweden (S2). An original study in which the BCS of SACs was the subject could not be found at the time of the literature search. For further details, see Table 1 and Tables S1-S4.

#### Body condition scoring in alpacas and llamas from breeding, governmental and other organizations

Seven of all sources (20.0%) were from breeders (A1-5), governmental (A7) or other (A6) organizations. This group reflected the greatest geographic diversity, as these came from four different countries. Interestingly, there were two sources from New Zealand, one from a breeding organization (A1), the other from the government (A7), which used similar graphic illustrations, but were not identical in content. On the other hand, a British association (A5) used the same content score sheet as an Australian association (A2). For more detailed information, see Table 1 and Tables S1-S4. None of these sources was found via the scientific databases, but via Google search or the snowball system.

#### Body condition scoring in alpacas and llamas from veterinary services

Seven of the sources (20.0%) were from veterinary services (V1-7). Of these, three were from the UK (V1,5,6), one from a larger association of livestock veterinarians (V6), one from a livestock practice (V5), and one from a practice specialized in camelids (V1). Three were from a practice specialized in camelids from Australia (V2-4), and another from a College of Agricultural Sciences in the USA (V7). Of these seven sources, only one includes llamas (V7); the other six sources are exclusively about alpacas (V1-6). As with the sources on breeding, governmental and other organizations (A1-7), none of these sources was found in the scientific databases, but only in the Google search and through the snowball system.

#### Other sources that dealt with a BCS in SACs but did not meet the inclusion criteria of this study

Google search and snowball search identified additional sources that dealt with BCS in SACs but did not meet the inclusion criteria of this study. Not included were sources in other languages than English (Frezzato and Stelletta n.g.; Wagener and Ganter 2020; Gauly 2018; Franz 2017; Trah and Wittek 2013), descriptions of a BCS for SACs that did not specify a numerical classification of scores (Rockett and Bosted 2007); scores published by companies, individual breeders, or private individuals (PMI Nutrition International 2021; Textile Exchange 2021); and scores for which no clear information about the origin could be provided. A thesis investigating the BCS in alpacas (Whittle 2015) was also not included in the evaluation, as it was stated that it used a previously described method (V7) for assessing the BCS.

### Authors

Some authors were involved in several descriptions for assessing a BCS in SACs: Van Saun is listed as author of six (17.1%) descriptions (T7-9, S9-10, V7), Vaughan of three (8.6%) sources (V2-4), and Johnson of two (5.7%) descriptions (T4, S5). The other authors each appeared once. However, it was only possible to make a clear statement about the scores from the textbooks, the scientific publications, or the veterinary services, as the descriptions of the associations (except A4) did not include specified author names.

### Geographic and temporal distribution of the sources

The sources were from five different countries. More than half of the descriptions of a BCS in alpacas or llamas were from the USA (57.1%; *n* = 20: T3-10; S1,3–11; A6,7), seven (20.0%: T1,2; A5,6; V1; V5,6) were from the UK, five (14.3%: A2,3; V2-4) were from Australia, two (5.7%: A1,7) were from New Zealand, and a single description of a BCS (2.9%: S2) was from Sweden.

**Fig. 2:**
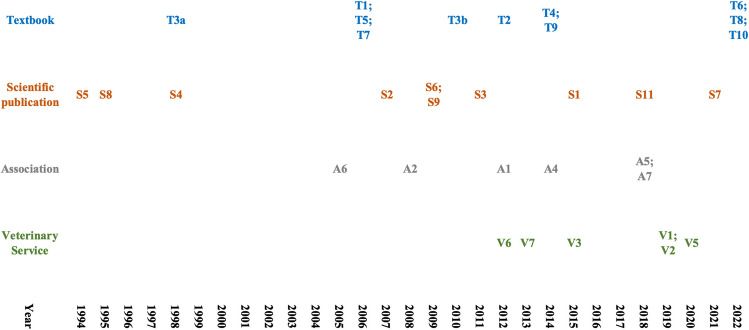
Chronological overview of sources that include a description of the BCS of South American camelids. Only 32 of the 35 sources are shown in this figure, as no information about the date of publication was available for three of the sources. Further details can be found in Table 1 and Tables S1-S4

The time span of publication of the sources was 28 years (1994–2022). However, the number fluctuated over the years with a sharp increase in the mid 2000s. Only three of the evaluated sources (8.6%; S4, 5,8) were from the 1990s, nine (26.5%; T1,3,5,7; S2,6,9; A2,6) were from the period 2001–2010, and almost half of the investigated sources (51.4%; *n* = 18; T2,4,9; S1,3,11; A1,4,5,7; V1-3,5–7) were from the period 2011–2020 (Fig. 2), four (11.4%) were from 2021 (S7) or 2022 (T6,8,10). For three sources (8.8%: S10; A3; V4), no chronological classification was possible. While the number of sources from scientific papers was more or less constant over the investigated three decades, sources from associations first appeared in the mid 2000s (A6), and the first description of a veterinary service was not found until 2012 (V6). The textbooks must be considered with caution in this evaluation, since only the most recent edition was included in the study. In addition, the dates of the sources from the associations and veterinary services were not necessarily accurate, as these were usually online sources and it was not always clear whether earlier versions of the descriptions existed.

### Species

Nineteen sources (54.3%: T1,2,4,6–8,10; S1,3,6,7,9–11; A4,6; V7) described the BCS for both lamas and alpacas, 13 sources (37.1%: T5,10; S2; A1-3,5; V1-6) each mentioned only alpacas in the description and four sources (11.4%: T3; S4,5,7) only llamas. Taking this together, 31 of the sources (88.6%) dealt with alpacas and 22 (62.9%) dealt with llamas.

None of the sources described the extent to which the examination should be assessed differently between alpacas and llamas. It was noticeable that all sources from the USA except one (T5) included llamas, while all sources from outside the USA described alpacas but contained llamas less frequently (only T1,2; A4,7). Furthermore, a temporal trend between the relevance of llamas and alpacas also seems to be apparent: while all descriptions from the 1990s were for llamas only, alpacas were represented in all descriptions from 2011 onwards.

### Practical examination of the BCS

**Fig. 3:**
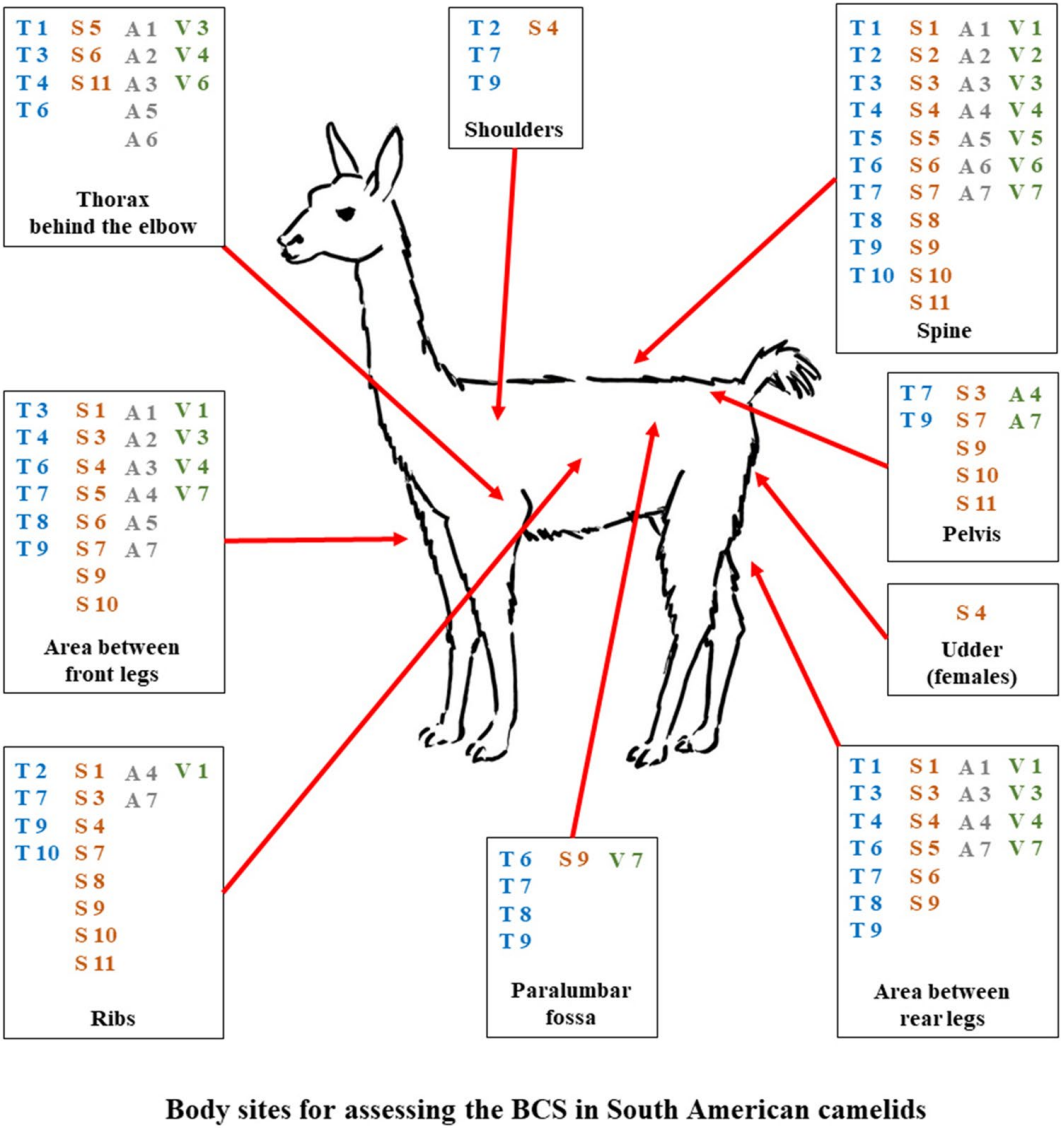
Body sites for assessing the BCS in alpacas and llamas. Overview of the body sites for the BCS assessment recommended by the different sources. T(1–10): textbooks; S(1–11): scientific publications; A(1–7): associations; V(1–7): veterinary services. See Table 1 or Tables S1-S4 for references. Schematic Drawing © L. Grimm

For the practical assessment of the BCS, different body sites were described in the sources. Those were usually examined by palpation, but in some cases a visual examination was indicated. The body sites found in the sources included the spine (lumbar region or withers), thorax, ribs, shoulders, pelvis, the paralumbar fossa, the area between the front legs, the area between the hind legs, and the udder in female animals. Depending on the source, at least one, but often several of these body regions were examined when assessing a BCS in alpacas or llamas (Fig. 3).

#### Spine

Palpation of the spine was the most important body site for assessing a BCS in SACs, as it was recommended in all 35 sources. Five sources (14.3%: T3,5,6; S8,11) suggested examining the withers. All sources suggesting an examination of the withers were from the USA; four of these recommended palpation of the withers (T3,6; S8,11), while the fifth source suggested palpation of the spinal area 15 cm behind the withers (T5).

The exact focus that should be set when examining the lumbar region varied between the sources; the backbone near the last rib (A2,3,5; V2-6), the dorsal or transverse processes (T2,7,9; S1,2,4,8), or the mid back (T4,10; A6; V1) are mentioned. Further details on the exact examination of the spine within the different scoring systems can be taken from Tables S1-S4.

#### Ribs/thorax behind the elbow

The examination of the ribs or thorax was mentioned by all but six of the investigated sources (17.1%: T5,8; S2; V2,5,7). The sources that included the thorax in their examination can be further divided into two groups. About half of these, i.e., 15 sources (42.9%: T1,3,4,6; S5,6,11; A1-3,5,6; V3,4,6) explicitly described that the thorax should be palpated at the point behind the elbow, while a further 15 sources (42.9%: T2,7,9,10; S1,3,4,7–11; A4,7; V1) only mentioned the palpation of the "ribs" without specifying this more precisely. One source (S11) mentioned both ribs and ribs behind the elbow. All sources stated that the examination should be done by palpation. Further details can be found in Table 1 or Tables S1-S4.

#### Shoulders

Palpatory examination of the animal's shoulders was suggested by a total of four sources (11.4%), three of which were from the USA (T7,9; S4) and one from the UK (T2). Descriptions by the same authors varied in terms of assessing the shoulder or not. In two sources where van Saun was involved as an author and which are very similar in content, the examination of the shoulder was mentioned (T7,9). In the other two sources where van Saun was author, the examination of the shoulder was not mentioned (S9; V7).

#### Pelvis

When comparing the information for the examination of the pelvis in the investigated scoring systems, contradictory information can be found. The 35 sources can thus be divided into three groups: scoring systems that advised pelvic examination, scoring systems that explicitly advised not to examine the pelvis, and scoring systems that did not provide any information on pelvic examination (Table 1 or Tables S1-S4). A total of nine sources (25.7%: T7,9; S3,7,9–11; A4,7) advised palpatory examination of the pelvis. Of these sources, seven were from the USA (T7,9; S3,7,9–11), four of which van Saun was involved as author (T7,9; S9,10), one source each came from the UK (A4) and New Zealand (A7). However, 13 sources (37.1%: T2-4,10; S2,4,5; A2,3,5; V3,4,6) from four different countries advised explicitly against palpation of the pelvis. The reason given was that this region of the body was not very representative, since even fat animals felt lean on the pelvic bones. In the remaining 13 sources (37.1%: T1,5,6,8; S1,6,8; A1,6; V1,2,5,7), there was no information on the examination of the pelvis. Some authors have turned away from examining the pelvis in their more recent descriptions. In two recent sources, where Van Saun was involved, the examination of the pelvis was not mentioned (T8; V7). In the information from the British Alpaca Society, the examination of the pelvis was recommended in an older description (A4), whereas the palpation of the pelvis was discouraged in the body condition scoring in the most recent description (A5).

#### Area between front or rear legs

The examination of the area between the front legs was suggested by 24 sources (68.6%: F3,4,6–9; S1,3–7,9,11; A1-5,7; V1.3,4,7), and the area between the hind legs by 21 sources (60.0%: T1,3,4,6–9; S1,3–6,9; A1,3,4,7; V1,3,4,7). Thereby, 20 sources (57.1%: T3,4,6–9; S1,3–6,9; A1,3,4,7; V1,2,7) recommended the examination of both the front and the back legs. Three sources (8.6%: S7,11; A2) recommended only examining the area between the front legs, and only one source (2.9%: T1) recommended only examining the area between the rear legs.

Although most sources indicated that nutritional status in SACs should be assessed palpatorily, in the case of the areas between the front and rear legs also visual examination was advised by some of the sources. Concerning the examination of the area between the rear legs, 11 sources (31.4%: T1,7; S3-5,9;A1,4,7; V1,7) advised performing only a visual examination, three (8.6%: T9; V3,4) advised a combination of palpatory and visual examination, and three others (8.6%: T3, S1,6) advised a palpatory examination of this area alone. In four of the investigated sources (11.4%: T4,6,8; A3), it was unclear from the description whether a palpatory or visual examination of the area between the rear legs should take place. When examining the area between the forelegs, seven of the scoring systems (20.0%: T7; S3-5,9; V1,7) advised visual assessment alone, eight (22.9%: T9, A1,2,4,5,7; V3,4) advised a combination of palpatory and visual examination, and five (15.3%: T3; S1,6,7,11) advised a palpatory examination of this area alone. In four sources (11.4%: T4,6,8; A3), it was unclear from the description whether a palpatory or visual examination of the area between the rear legs should take place.

#### Fossa paralumbalis

The evaluation of the paralumbar fossa was proposed only in six sources (17.1%: T6-9; S9; V7), that were all from the USA; in all but one source (T6) van Saun was author of these descriptions. There were no precise instructions on how the examination should take place, only schematic illustrations served as orientation.

#### Udder (in female animals)

Only one source (2.9%: S4) from the USA from the 1990s recommended visual assessment of the udder when evaluating rear legs.

### Accuracy of the descriptions for BCS assessment in SACs

Descriptions of the practical procedure for assessing a BCS in SACs varied in accuracy depending on the source. Less than half of the investigated sources (48.6%; n = 17: T3-6,S1,5,6; A1-5,7; V1,3,4,7) gave a certain order of examination, whereas in 16 sources (45.7%: T1,2,7–9; S3,4,8–11; A6; V2,5–7) no exact order of examination was suggested. In two sources (5.7%: T10; S2), only one body site was examined, so that an order of examination is not applicable here.

A total of 20 sources (57.1%: T1,5,7–9; S2,9,10; A1-5,7; V1-4,6,7) provided a description for each score, which were usually presented as schematic illustrations. Especially the sources from the associations and veterinary services contained quite precise descriptions. The other 15 sources (42.9%: T2-4,6,10; S1,3–8,11; A6; V5) gave no exact description for every score. In this group, the scientific publications were overrepresented: eight (S1,3–8,11) of eleven scientific publications did not provide detailed descriptions of each score.

### Scales

The BCS in the investigated scoring systems was classified using a scale with numbers in ascending order as in other species (Kenyon et al. 2014; Panne and Mansfeld 2022). The lowest value of the scale described an emaciated animal; the highest value of the scale described an obese animal. In the 35 investigated sources, a scale from 1–5 was used most frequently (26 times; 74.3%: T2,5–9; S1-3,7–11; A1-5; V2-7); ten scoring systems (28.6%: T1-3,6; S4-6,11; A6; V1) used a scale from 1 to 10 and five scoring systems (14.3%: T4,9,10; S1,10) used a scale from 1–9. A scale from 0 to 5 was mentioned only once (2.9%: T1). The scale from 0–5 may also be a typographical error, since 0 was not mentioned anywhere else in the text of the source and 1 was given as the lowest score value in the description (T1). Seven scoring systems (20.0%: T1,2,6,9; S1,10,11) described two different scales.

Geographic differences were noticeable in the distribution of the scales that were used for body condition scoring. All the scoring systems that used a higher scale and gave a score from 1–9 or 1–10 came from the USA (T6,9,10; S1,10,11; A6) or the UK (T1,2; V1). The scoring systems from Sweden (S2), Australia (A2,3; V2-4), and New Zealand (A1,7) exclusively used a scale from 1–5.

### Ideal BCS

All but four sources (11.4%: T2,8; S8,10) specified an ideal BSC. Of these 31 sources, 14 (40%: T3,4,6,10; S1,2,4–7,11; A6; V1,2) gave a specific value or range of values as the optimal BCS for all animals regardless of age or other influencing factors. The other 17 (48.6%: T1,5,7,9; S3,9; A1-5,7; V3-7) gave possible physiologic deviations in addition to the optimal BCS. There was an imbalance between the four groups of sources. While most of the scoring systems from associations or veterinary services considered several influencing factors in this respect, that was the case in less than half of the textbook sources (T1,5,7,9) and in only two of 11 (18.2%) scientific papers (S3,9).

Influencing factors that were mentioned were the breed (huacaya or suri), age, sex, pregnancy, lactation, and the season. In principle, the ideal score was in the middle of the scale with around 3 for scales up to 5, and around 5 for scales up to 9 or 10. In three scoring systems (8.6%; two from the UK (T1; V1), one from the USA (A6), an ideal score was given slightly above the median, whereas the optimal BCS in three sources (8.6%: V2-4) that were all from a veterinary service in Australia and published by Vaughan was given as just below the median.

#### Species

In none of the sources was there any reference to the difference between alpacas and llamas concerning the BCS, in more than half of the sources the score was described for both species.

#### Breed

Three of the sources (8.6%), two from the associations (A1,4) and one from the veterinary services (V1), made a statement about the breed: adult huacaya alpacas should have a BCS of 3 (scale of 1–5), but adolescent huacayas under one year of age should have a BCS of 4 (scale of 1–5) (A1,4), suris should have a BCS of 4–5 (A4) or at least 5 (scale of 1–5) (A1), as suris tend to store more fat reserves around the backbones (V1). However, there was no information about different types of llamas.

#### Age

A total of nine sources (25.7%: T5; A1-4,7; V4,6,7), all but one from the associations or veterinary services, provided information on age influencing the ideal BCS. According to those, the ideal BCS of 3 (scale of 1–5) applied to alpacas over two years of age (A4,7). Growing alpacas < 15 months should have a BCS of 3–3.5 (scale of 1–5) (A2,3; V7), adolescent huacaya alpacas under 1 year of age should have a BCS of 4 (scale of 1–5) (A1,4), and crias under 6 months of age should have a BCS of 5 (scale of 1–5) (A1,4). In two sources, "growing" was not further specified; the ideal BCS for this group of animals was mentioned as 2.5–3 (scale of 1–5) (V4) or 2.5–3.5 (scale of 1–5) (V7). Furthermore, it was stated that older alpacas often also had a BCS of 2 (scale of 1–5) (T5). One source further stated that older alpacas felt different than younger ones and that palpation of the ribs was especially important in older animals (V1). There was no information in the sources explicitly about the influence of age on the BCS in llamas.

#### Sex, pregnancy, and lactation

In some sources from Australia or the UK, the males were further divided into whether they were non-working or working males (11.4%; *n* = 4: A2,3; V4,6) (Table 2). The differences here were not particularly large, optimal scores of 2.5–3.5 (scale of 1–5) were given. Only one source (V4) indicated that working males should have a higher BCS. None of the sources from the USA made a specific statement about males in this regard. For females, there were distinctions made between non-pregnant and pregnant ones, whereby pregnant females should generally have a higher BCS than non-pregnant females (Table 2). The values were usually quite similar with scores of 2.5–3.5 (scale of 1–5) given for non-pregnant females (S3; A2,3; V6) and 3.0–3.5 (scale of 1–5) for pregnant females (S3,9; A2,3; V6). The BCS of a dam at birth should be 3.0 (scale of 1–5) (V3,4). However, a comparison with pregnant females was not given in these sources. Six of the investigated sources (17.1%: T7,9; S3,9; V4,7) described a physiologic decrease in BCS during lactation. Two of those sources (5.7%: V4,7) indicated the value to which the BCS can decrease and four sources (11.4%: T7,9; S3,9) how many score points the BCS decreased. These were given with values of 0.5–0.75 (scale of 1–5) (T7); 0.75 (scale of 1–5) (S3,9) and 1.0 (scale of 1–5) (T9). The minimum score to which a lactating mare should drop was given as 2.25 (scale of 1–5) (V4) and 2.5–3.0 (scale of 1–5) (V7). It was surprising that those values are also given in increments of 0.25, while in the description of the scores, there were only increments of 0.5.

**Table 2:** Information on BCS regarding sex, pregnancy, and lactation. All data in the table refer to a scale from 1–5

Source	Country	Most animals	Growing	Wether	Mature male	Working male	Non-pregnant mature female	Pregnant female	Dam at parturition	Loss in Lactation
T7	USA	2.5–3.25						3.25–3.5		max 0.5–0.75
T9	USA	3						3.5		max 1.0
S3	USA	2.5–3.5					2.5–3.5	3.0–3.5		max 0.75
S9	USA	2.5–3.5						3.0–3.5		max 0.75
S10	USA	2.5–3.5						3.0–3.5		max 0.75
A2	Australia		3.0–3.5	2.5–3.5	2.5–3.5	2.5–3.5	2.5–3.5	3.0–3.5		
A3	Australia		3.0–3.5	2.5–3.5	2.5–3.5	2.5–3.5	2.5–3.5	3.0–3.5		
A5	UK		3.0–3.5	2.5–3.5	2.5–3.5	2.5–3.5	2.5–3.5	3.0–3.5		
V3	Australia	2.5–3.0	3.0			2.5–3.5			3.0	
V4	Australia			2.5	2.5	2.5–3.0	2.5		3.0	down to 2.25
V6	UK		3.0–3.5	2.5–3.5	2.5–3.5	2.5–3.5	2.5–3.5	3.0–3.5		
V7	USA	2.5–3.5	2.5–3.5					3.0–3.5		down to 2.5–3.0

### Frequency of examination

A total of 24 sources (68.6%: T1,3,5–9; S1,3,4,6,7,9–11; A1,3–6; V1,2,6,7) provided information on how frequently a BCS should be recorded in SACs. Of these sources, different examination frequencies were suggested. Eleven sources (31.4%: T1,3,5,8,9,11; A1,4,6; V2,6) indicated the frequency as "regular" or “periodically”. Various specifications were made, such as "at least twice a year" (A6), "regular weighing or BCS as an alternative" (T1,3; A4), regular for animals over 3 months" (T5) or "regular, especially during pregnancy and lactation" (S9). The other 13 sources gave specific time periods when the investigation should take place. In detail, the following suggestions were made for adult animals: twice a year (A6); at least six times per year (S4); every 4- 6 weeks (T7,9); monthly (S1,3,6,7; V1); “whenever handling the animals” (A1-3,5). Thereby, nine sources advised paying special attention to certain groups of animals (26.5%: T5-7,9; S1,3,9,10; V7) where “pregnancy”, “lactation”, or “systemic diseases” were mentioned. Furthermore, there was additional advice for juvenile animals; crias should be examined daily or every other day for the first two weeks (S1). In two of the sources (5.7%: S2,8), the BCS was described exclusively for a scientific experiment, so these did not provide any information about the frequency of the assessment on farms.

### Illustrations in the descriptions

All but four of the 35 sources (11.4%: T2; S3,7,8) provided photographs or schematic drawings to illustrate the respective scoring system. Twenty-eight sources (80%: T1,3,5–9; S1,2,4–6,9–11; A1-7; V1-5,7) illustrated the assessment of the BCS using schematic drawings. A lumbar cross section was shown in 26 sources (74.3%: T1,3,5–9; S1,2,4–6,9–11; A1-5,7; V1-3,5,7), a schematic view of the front or rear legs was shown in 11 sources (31.4%: T1,3,6–9; S4,5,9,10; V7). A difference between the sources could be found; although all descriptions of the associations (A1-7) contained schematic illustrations, they were all limited to the spine. The area between the front or rear legs was mainly illustrated by the textbooks and scientific publications (T1,3,6–9; S4,5,9,10).

Several sources also used very similar schematic drawings. Sources where Van Saun was involved as an author used a detailed table with schematic drawings of frontal profile, rear profile, spinous to transverse process, and the paralumbar fossa at different scores (T7-9; S8; V7). The author referred in his description to scoring systems from cattle (Edmonson et al. 1989), and previous scoring systems for llamas (S4,5). A schematic drawing showing a cross section of the lumbar spine can be found in modified form in most of the descriptions from the associations or from veterinary services (A1-5,7; V2,3).

Photographic illustrations were only found in three of the investigated sources (8.6%: T3,4,10). All were featured in the textbooks, one (T3) included photographs of fat or emaciated llamas or alpacas, another source (T4) that considered the palpation of the pelvis to be incorrect showed a photo of palpation in the pelvis as a misleading site for assessing body condition in alpacas. The third picture (T10) seems to have been taken on the same occasion as the photo from (T4), but here the correct palpation of the mid-back was shown.

Whitehead from "Camelid Veterinary Services" "also provided a youtube video that is freely available on the internet and very clearly and extensively highlights body condition scoring in an alpaca and the relevant background (V1). Another youtube video explaining the BCS in alpacas was provided by the “British Alpaca Society” (British Alpaca Society 2023).

### Assessment of BCS versus weighing

Whether Body Condition Scoring or weighing the animal is a better method of assessing the nutritional status was answered differently by the investigated sources. A total of 18 sources (51.4%: T1-3,5,6,8; S1,4,6,7; A2-7; V1,4) made a statement regarding the weighing of the animals. Eight sources (22.9%: T3,8; S1,4,7; A4,6; V1) saw both weighing and assessing a BCS as adequate methods. However, two of those sources indicated that for crias the body weight was a more meaningful parameter than the BCS (A4,7), and two other sources indicated that the BCS assessment should be done in addition to weighing the animal (T3, S1). Only two sources (5.7%: T1,2), both textbooks from the UK, saw weighing as a more suitable method, whereas four of the investigated scoring systems (11.4%: T5,6; A2,3,5; V4) saw the BCS as a better method than weighing. Three sources (8.6%: S6; A4,7) made a differentiated statement regarding this question, according to which weighing was of greater importance in crias, and the BCS assessment in adult animals. One other source emphasized the advantages (simple, low costs) of body condition scoring (V4).

## Discussion, Conclusion, and Outlook

The systematic literature search revealed 35 different descriptions for assessing a body condition score in SACs. While the first scores from the 1990s had a stronger focus on llamas, the focus in more recent years has been on alpacas. Almost 90% of the scores were described for alpacas, about 60% of the scores were directed at llamas. However, no clear distinctions were made between the two species, which is supported by the fact that more than half of the sources provided the BCS for both species. For the practical examination, it is common sense that the palpatory assessment of the BCS is preferred. All sources advised carrying out a palpatory examination of the spine, usually the lumbar vertebrae. The majority of them further advised an examination of the ribs, with some indicating the site caudal to the elbow as the precise location.

Almost 70% of the sources further indicated that the area between the front and rear legs should be examined. However, there was no consistent approach in those sources. There were suggestions for both palpatory and visual examination. Whether palpatory examination at these body sites is possible in every animal remains questionable, as does the additional benefit of examining these body sites. The practical implementation and utility of these examinations should be investigated in more detail.

The body site that was discussed most controversially in the sources was the pelvis. While some authors integrated this body site into the examination, others explicitly advised against it. Unfortunately, it was not clear from the sources how the pelvis should be examined in detail. Probably the palpation of *Tuber coxae* was meant, then the arguments would seem plausible that the rather bony haptic of the pelvis would lead to falsely low BCS (Johnson 1994). The fact that even authors who described the palpation of the pelvis earlier refrained from doing so in more recent descriptions should be a reason not to include the pelvis in the assessment of the BCS of alpacas and llamas. Body sites such as shoulders, paralumbar fossa, or udder were only occasionally mentioned. Furthermore, there was hardly any information on the practical assessment and the interpretation of the results. Therefore, these body sites can probably be ignored.

Altogether, the literature points to spine and thorax as the most essential body sites for body condition scoring in SACs. Whether a certain order has to be observed in the examination when only two body sites are considered seems questionable. A concrete description of each individual score, which can be found in more than half of the sources, is a useful aid for the handling and training of inexperienced examiners; the schematic drawings of the different scores are particularly helpful. Since different scales are used, confusion can quickly arise if the range of the scale is not explicitly stated. Similar problems with different scales are also known in cattle (Morris and Kenyon 2002; Garnsworthy 2006). For body condition scoring in SACs we suggest using only the most common published scale from 1–5, with 1: emaciated, 3: optimal, 5: obese, and then to use increments of no more than 0.5 steps in the future to avoid confusion due to different scales. On this scale, the ideal BCS is 3; however, physiologic influences due to breed, age, sex, pregnancy, and lactation have to be taken into account. Some of the information in the sources did not seem plausible: the optimal BCS for suris and crias was sometimes given as 5 on a scale from 1–5 (Alpaca Association New Zealand 2012; Turner 2014), without giving more detailed information. Suris tend to store more fat reserves around the backbones (Whitehead 2019), resulting in high BCS. This highlights the need to develop a separate score for suris and crias, or to classify the findings on spine and thorax in this breed and in newborns in such a way that the optimal score would again be 3 and therefore in the middle of the scale. A score with an optimal value at the upper end of the scale does not constitute a practical tool to assess variations.

Differences between species were not addressed in the sources, but two descriptions of a BCS in SACs, which were not included in this evaluation because they were published in German, indicated a difference in the ideal BCS between alpacas and llamas; according to both, the optimal BCS should be 2–3 (scale of 1–5) for alpacas and 3 (scale of 1–5) for llamas (Verein der Züchter, Halter und Freunde von Neuweltkameliden e. V. 2015; Gauly 2018).

All of the investigated sources encourage a regular assessment of the BCS; a pragmatic approach can be found in the suggestion "whenever handling the animals" as this does not involve additional effort or stress to the animals. A monthly assessment was the most commonly cited time interval in the available literature. Single groups of animals should also receive special attention, including females in pregnancy or lactation or sick animals (Table 2). In newborn crias, control of body weight gain seems to provide better information than a BCS, at least for the first two weeks of life. New-born alpacas weigh 6–9 kg, new-born llamas 8–18 kg (McLean and Niehaus 2022). However, there is different information on daily weight gain of crias in the literature. Whereas Whitehead states that after an initial weight loss of approx. 250 g on the first day of life, daily weight gains of 250–500 g can be expected in crias (Whitehead 2009), Gauly indicates a daily weight gain of 120 g for alpaca crias and 200 g for llama crias (Gauly 2018).

The question whether BCS or body weight provides a better parameter for the nutritional status of the animals could not be clarified by this literature review. Although there were more sources stating that the BCS is a better parameter, some sources rated the body weight as being better. Both methods have advantages and disadvantages (Gauly 2018). If body weight is regularly checked and documented, it is an objective parameter that can provide a trend of the animal's nutritional status. However, the degree of filling of the compartments, and, in the case of advanced pregnancy, the proportion of the fetus should also be taken into account (Gauly 2018). A single body weight, on the other hand, has only limited significance, as body weight is not necessarily correlated with the BCS of an animal (Wildman et al. 1982). Depending on the phenotype and age of the animal, the single value does not provide a comprehensible estimate of the nutritional status, especially in growing animals. When an alpaca or llama is examined for the first time, for example, when an animal is presented for veterinary examination, the BCS provides a quick impression of the nutritional status without the need for any other equipment.

Despite the fact that the BCS has been standardized, it will always remain a subjective assessment (Kenyon et al. 2014). In cattle and sheep, there are studies that calculated the inter- and intra-rater reliability of the BCS (Kristensen et al. 2006; Kenyon et al. 2014; Corner-Thomas et al. 2020; Phythian et al. 2012). In a recent study, we assessed the inter-rater reliability for the BCS of llamas and alpacas due to palpatory examination of the lumbar vertebrae and found that it was comparable to that of cattle and sheep (r = 0.52–0.89; τ = 0.43–0.80; κ_(w)_ = 0.50–0.79) (Wagener et al. 2023).

Even though there is already a lot of information about the BCS in SACs, some open questions remain. Firstly, the intra-rater reliability (de Raadt et al. 2021; Phythian et al. 2012) has not yet been investigated for the assessment of BCS of alpacas or llamas. It is unknown how exactly single examiners can reproduce their results when assessing the BCS. Secondly, it is still not agreed upon which body sites are essential for including in the assessment of a reliable BCS in SACs. Besides the spine, additional body sites were presented in different sources, but these sources provided only superficial information on the interpretation and significance of the findings obtained with those parameters like the ribs or the paralumbar fossa. Zielke et al. found that inter-rater reliability of BCS in bison vary depending on the assessed body site (Zielke et al. 2018).

In addition, a more detailed characterization of the BCS should be performed for different breeds. It implied that Huacaya and Suri alpacas have to be evaluated differently due to their anatomy (Alpaca Association New Zealand 2012; Turner 2014), but there was no information on the different llama types, or even the difference between alpacas and llamas.

This scoping review, like any scientific work, must be interpreted with some limitations. Only the English literature was evaluated. Available descriptions in other languages were not taken into account within the results, but were partly included in the discussion. On the other hand, some authors were involved with more than one source. For some sources, no person could be identified as a clear author, so that a bias could arise here due to the overrepresentation of individual opinions. Furthermore, some sources included no references, leaving the possibility of reproduced information from other sources.

In summary, the scoping review showed that the BCS is a widely used tool in husbandry and veterinary medicine for alpacas and llamas. Due to the risk that changes in nutritional status may remain hidden for a long time given the dense fiber coat of the animals, palpatory examination of the animals is a simple and quick method of diagnosis. To date, the BCS has not been clearly standardized and a great deal of the information available in the investigated sources is based on experience rather than scientific evidence. Further studies should be carried out to prove the actual significance of the BCS in alpacas and llamas. In particular, the inter- and intra-rater reliability of the BCS at the different presented body sites should be investigated individually. Further correlations between the BCS and body weight in animals of different groups, such as working males or pregnant mares, should be examined.

### Supplementary Information

Below is the link to the electronic supplementary material.

Supplementary file1 (PDF 242 KB)

## Data Availability

All relevant data are included in the manuscript.
