# Supplementary material for: Body condition scoring in alpacas (Vicugna pacos) and llamas (Lama glama) – a scoping review
Source: Vet Res Commun. 2023 Dec 22;48(2):665–84. doi: 10.1007/s11259-023-10275-y (PMC10998785; doi:10.1007/s11259-023-10275-y)
Supplement: Supplementary file 1 — Supplementary file1 (PDF 242 KB) [file 11259_2023_10275_MOESM1_ESM.pdf]

Body Condition Scoring in alpacas (*Vicugna pacos*) and llamas (*Lama glama*) – a scoping review

Matthias Gerhard Wagener [1*], Martin Ganter [1] and Sabine Leonhard-Marek [2,3]

[1]Clinic for Swine, Small Ruminants, Forensic Medicine and Ambulatory Service, University of Veterinary Medicine Hannover, Foundation, Hannover, Germany

[2]Library, University of Veterinary Medicine Hannover Foundation, Hannover, Germany

[3]Department of Physiology, University of Veterinary Medicine Hannover Foundation, Hannover, Germany

E-mail: Matthias.gerhard.wagener@tiho-hannover.de

Supplementary Material: Tables S1-S4

Table S1: Detailed overview on sources about body condition scores for alpacas and llamas from textbooks about llama and alpaca medicine or husbandry (sorted alphabetically by first author). If more than one edition were available for a textbook, the most recent edition was taken into account. A: alpaca; L: llama.

Source	Author	Edition	Reference	Country	Year	Species	Scale	Description
T1	Bromage	1	(Bromage 2006)	UK	2006	A;L	0-5; 1-10	The BCS is described in a textbook about llama and alpaca management in the chapter "Feeding and Nutrition". The author considers regular weighing of the animals as a more accurate method and assessing the BCS only as an alternative. The evaluation of the BCS is described as a hands-on method, where loins and down the ribs at the side of the chest should be palpated. In lightly fibred animals, the visual assessment of the area between the hind legs can also be included in the evaluation of the BCS. The description is supplemented by three schematic illustrations showing the examined body sites, as well as cross sections of the lumbar spines, and the rear view of an animal for each of the scores 1-5. The ideal score is given as 3.5 to 4 out of 5.
T2	Duncanson	1	(Duncanson 2012)	UK	2012	A;L	1-5; 1-10	The BCS is described in a textbook for veterinarians in the chapter "Nutrition and Metabolic Diseases". The author recommends body weight check every two months, a preference between BCS or body weight is not indicated. The description also points out the different scales commonly used (1-5 and 1-10). The BCS should be determined by palpation of transverse processes of the lumbar vertebrae, the shoulders and the loin. The author points out that the pelvic bones are easy to feel in SAC

								because fat accumulates first in the brisket area, between the hindlegs and around the peritoneum. In another chapter of the textbook the BCS is included in a checklist for the evaluation of the phenotype. An ideal score is not specified separately.
T3	Fowler	2;3	(Fowler 1998);(Fowler 2010)	USA	1998; 2010	L	1-10	The BCS is described in the second and third edition of a veterinary textbook about camelids in the chapter “Feeding and Nutrition” in the same way. The author recommends weighing the animals regularly, the assessment of the BCS is considered as second choice. The investigation is based on Johnson (1994) (S5) by observing and palpating the withers, the fiberless areas behind the elbow, between the rear legs, the chest and the perineum in the mentioned order. The description is supplemented by several schematic illustrations showing the individual examination sites, cross-sections of the spinous processes, and the front and rear views of an animal. Also included are photos of thin and fat llamas. The ideal score is given as 5.
T4	Johnson	1	(Johnson 2014)	USA	2014	A;L	1-9	The BCS is described in the chapter "Physical Examination and Conformation" in a veterinary textbook about camelids. The score is described as an important subjective component of the physical examination. The body parts to be examined are the mid back and the thorax behind the point of the elbow. The author warns not to palpate the pelvis, because all camelids feel bony and a too low BCS could be detected, this statement is supported by a photographic illustration. The ideal score is given as 5.

T5	McConnell and Hoffman	2	(McConnell and Hoffman 2006)	USA	2006	A	1-5	The BCS is covered in the chapter "On-Farm Assessment and Diagnosis" in a textbook about alpacas. The authors consider BCS as a more accurate method compared to weighing, they point out different scales in common use, but recommend using a scale from 1 to 5. The examination is performed only by palpation of the spine 15 cm behind the withers. This description is supplemented by schematic illustrations. The BCS should be assessed regularly in adults, but this score is not appropriate for crias under 3 months of age. An ideal score is given as 3.
T6	Niehaus	4[a)]	(Niehaus 2022)	USA	2022	A;L	1-5; 1-10	The BCS is described in the fourth edition of a veterinary textbook (formerly T3). in the chapter “Physical Exam and Diagnostics”. The author considers body condition scoring to be more appropriate than weighing for assessing the nutritional status of the animal. The author refers to Johnson (1994) (S5) and Van Saun (2013) (V7). The withers, the fiberless areas behind the elbow, between the rear legs, the chest and the perineum should be examined, however, there is no clear statement as to whether the examination should be performed by observation or palpation. Body condition scoring should be performed especially during pregnancy, as well as between early and mid-lactation. the descriptions are supported with the same schematic drawings as in Fowler (2010). The author gives 5 (at 1-10) as the ideal BCS.
T7	Van Saun	2	(Van Saun 2006)	USA	2006	A;L	1-5	The BCS is described in the chapter "Feeding the Alpaca". The author refers to Edmonson et al. (1989) (cattle) (Edmonson et al. 1989), Russel (1991) (sheep), Johnson (1994) (S5) and Hilton et al. (1998) (S4). The author

								points out the different scales used, suggests the use of a score from 1 to 5 (with subdivisions of 0.5) in connection with a very detailed schematic drawing. The examination of the BCS is described as a hands-on method in which the subcutaneous fat deposits on ribs, shoulder, loin and pelvis are examined by palpation. The BCS examination should be performed regularly (4 to 6 x / year) and especially during early to mid-pregnancy, as well as early to mid-lactation. The ideal score is given as 3.0. Most animals in a herd should have a BCS of 2.5 to 3.25. Animals in late pregnancy should have a BCS of 3.25 to 3.5, the BCS of animals in lactation should decrease by a maximum of 0.5 to 0.75.
T8	Van Saun	4[a)]	(Van Saun 2022)	USA	2022	A;L	1-5	The BCS is described in the chapter “Feeding and Nutrition”. The author refers to Hilton et al. (1998) (S4) and Van Saun (2013) (V7). The author recommends regular weighing of the animals or assessing the BCS. The practical examination of the BCS should be done by observing and palpating of different regions of the body. For this purpose, the author refers on the one hand to the schematic drawing given in the chapter "Physical Exam and Diagnostics" in the same textbook, which was already used previously by Fowler (2010) (T3). At the same time, the schematic drawing of Van Saun (2006) (T7) is given.
T9	Van Saun and Herdt	1	(Van Saun and Herdt 2014)	USA	2014	A;L	1-5; 1-9	The score is described in the chapter "Nutritional Assessment". The assessment of the BCS is seen as the most efficient subjectively method for evaluating the energy status of a nutritional program. The authors refers to Edmonson et al (1989) (cattle) (Edmonson et al. 1989),

								Russel (1991) (sheep), Johnson (1994) (S5) and Hilton et al. (1998) (S4). The authors points out the different scales used, suggests the use of a score from 1 to 5 (with subdivisions of 0.5) in connection with a very detailed schematic drawing (the same like Van Saun (2006) (T7)), but also give information about the score from 1 to 9. The examination of the BCS is described as a hands-on method in which the subcutaneous fat deposits on ribs, shoulder, loin and pelvis are examined by palpation. The BCS should be assessed mainly during early to mid-pregnancy and early to mid-lactation, but also regularly (4 to 6 times a year). The ideal score is given according to the scale used: 3 (scale from 1 to 5) or 5 (scale from 1 to 9); most animals in a herd should have a BCS of 2.5 to 3 (scale from 1 to 5) or 4 to 6 (scale from 1 to 9); animals in late pregnancy should have a BCS of 3.5 (scale from 1 to 5) or 6 (scale from 1 to 9), the BCS of animals in lactation should decrease by a maximum of 1 (scale from 1 to 5) or 2 (scale from 1 to 9).
T10	Wiedner	11	(Wiedner 2022)	USA	2022 b)	A	1-9	The BCS is described in the chapter “Management of Llamas and Alpacas” section “Feeding and Nutrition of Llamas and Alpacas” of the “MSD Veterinary Manual”. The examination is described as a hands-on method, by palpation of neck, lumbar vertebrae and the ribs. The author recommends not to palpate the pelvis, as this could lead to false thin impressions. The description is illustrated by a photo showing palpation of the backbone. The author refers to Johnson (T4; S5). The ideal score is given as 5.

a): This textbook is the fourth edition of the textbook (T3). However, since the author and editor of edition 1-3 (Fowler) had deceased in the meantime, the textbook was revised and now published by another editor (Niehaus). b): the latest 11th edition was released in 2016, the website indicates that this article was last modified in 2022.

Table S2: Detailed overview on sources about body condition scores for llamas and alpacas from scientific publications in an alphabetical order by first author. A: alpaca; L: llama.

Source	Author	reference	Country	Year	Species	Scale	Description
S1	Bennet and Richards	(Bennett and Richards 2015)	USA	2015	A;L	1-5; 1-9	The BCS is described in an overview paper on "Camelid Wellness". The authors refer to Van Saun and Herdt (2014)(T9). Different available scores are mentioned. Regular weighing as well as regular assessing of a BCS is advised, if possible from a constant assessor. To determine the BCS, the lumbar region should be palpated to assess the soft tissue and vertebrae. Furthermore, the fat deposits of the ribs and between the front and hind legs should be checked. The descriptions of the examinations are supplemented by schematic illustrations with cross-sections of the spine. Examinations should be performed daily or every other day for newborns during the first two weeks and monthly for healthy adults. An ideal BCS is not explicitly stated; a BCS of 3 (from 1 to 5) is considered moderate.
S2	Folkesson	(Folkesson 2007)	Sweden	2007	A	1-5	The BCS is described in the material and methods part of a thesis on feeding of alpacas in Sweden. The BCS was assessed weekly in the lumbar area for this study. Schematic illustrations with cross sections of the spine for each score are additionally given. The author points out that the pelvis should not be included in the study because overconditioned animals will also appear thin. For each score point there is a detailed description.
S3	Gomez	(Gomez 2011)	USA	2011	A;L	1-5	The BCS is described in a review paper on "Nutritional Assessment, Nutritional Requirements and Forage Analysis of Llamas and Alpacas". The assessment of the BCS is described as a hands-on method where ribs, loin, and pelvis for thickness of fat cover should be palpated. The area between the front and rear legs should be visually assessed. The BCS should be assessed once a month, especially when

							animals are sick, pregnant or in lactation. The ideal score is given as 2.5 to 3.5, and 3 to 3.5 in late pregnancy. the decrease in lactating animals should not exceed 0.75.
S4	Hilton et al.	(Hilton et al. 1998)	USA	1998	L	1-10	The description of the BCS can be found after a study of estimating the body weight of llamas by adspection, palpation and with the help of weight tapes. The authors refer to the BCS by Johnson (1994) (S5) and extend it. They recommend the palpation of transverse processes of the lumbar vertebrae, the shoulders, the ribs and the loins. The visual examination includes the regions between the rear legs and at the brisket, and the udder in female animals. They also advise not to include the pelvis in the assessment of BCS in llamas. The description of the assessment is supplemented by several schematic illustrations showing cross-sections of the loin and views of lamas from the front and back. The examination should be performed at least 6 times a year, the ideal score is given as 5.
S5	Johnson	(Johnson 1994)	USA	1994	L	1-10	The author presents the BCS in a review paper on llama nutrition, he sees body condition scoring as an important examination in the feeding management. The subjective score is determined by palpating the loin and the fibreless area behind the elbow and by visual examination of the regions between the rear legs and at the brisket. Schematic illustrations showing a cross section of the spine, as well as views of the animal from the front and back support the description. Furthermore, he advises not to include the pelvis in the assessment of BCS in llamas, as even very fat animals can feel bony there. The ideal score is given as 5.
S6	Jones and Boileau	(Jones and Boileau 2009)	USA	2009	A;L	1-10	The description of the BCS can be found in a review article about "Camelid Herd Health" in which different routine methods are described. The authors consider the examination of BCS after weaning

							at the age of 4 to 6 months to be more important than weighing. They point out that due to the thick fibre coat of the animals the examination must be performed by palpation. The loin and the thorax behind the elbow are palpated. In obese animals, the evaluation should also include the fat deposits between the front and hind legs. In addition, schematic illustration of a cross-section of the spine at three different scores are given. The BCS should be assessed monthly, but as it is a subjective method, it should be performed by the same person, if possible. The ideal score is given as 5.
S7	Lopez	(Lopez 2021)	USA	2022	A;L	1-5	The BCS is described in a review paper on veterinary management of adult camelids. The examination should be performed by palpation of ribs, hip bones and the keel of the sternum. The author recommends the assessment of the BCS or weighing of the animals monthly. The ideal BCS is given as 3.
S8	Morin et al.	(Morin et al. 1995)	USA	1995	L	1-5	The BCS is described in the materials and methods section of a research paper on the composition of milk from llamas in the USA. The BCS in this study was assessed by palpation of dorsal and transverse processes of the lumbar spine, ribs and withers with gradations of 0.5.
S9	Van Saun	(Van Saun 2009)	USA	2009	A;L	1-5	The BCS is described in a review paper on "Nutritional Requirements and Assessing Nutritional Status in Camelids". It refers to Johnson (1994) (S5), Hilton (1998) (S4) and Van Saun (2006) (T7). The author points out the subjectivity of the method and the different scales commonly used, recommending a scale from 1 to 5 (with subdivisions of 0.5). The schematic illustration from (T7) has been adopted. The BCS is described in the same way as for sheep, the fat deposits on ribs, loin and pelvis are to be examined palpatory, the area between the front and hind legs is to be examined visually. The BCS should be

							examined regularly, especially during pregnancy and lactation. The ideal score is 3.0, most animals in a herd should have a BCS of 2.5 to 3.5, animals in late pregnancy 3.0 to 3.5. During lactation the BCS should not decrease more than 0.75.
S10	Van Saun[1]	(Van Saun n.g.)	USA	n.g.	A;L	1-5	The BCS is described in a review paper on "Nutritional Overview: Forage, Feed, Minerals and Other Supplements". The author refers to Hilton et al. (1998) (S4) and Johnson (1994) (S5), and points out that the assessment is a subjective investigation. The loin, ribs, pelvis and should be palpated. The schematic illustration from (T7) has been adopted. Assessment of the BCS should be performed periodically, especially during pregnancy and lactation.
S11	Walker	(Walker 2018)	USA	2018	A;L	1-10 (1-5)	The BCS is described in a review paper on camelids published in proceedings for bovine practitioners. The examination should be performed by palpation, the backbone behind the shoulders at the withers, the ribs, the hip, the chest immediately behind the elbow, and brisket should be examined. The author proposes a scale of 1 to 10, but points out that 1 to 5 is also common. The description is supported by a schematic illustration of a vertebra with musculature in cross-section. The assessment of the BCS should take place regularly and is best done by the same person each time. The ideal score is given as 5 (1-10).

[1] whether this source was published in a scientific journal was not apparent. However, the structure of the source resembles that of a scientific review, which is why it was included in this category.

Table S3: Detailed overview on sources about body condition scores for llamas and alpacas from breeders, governmental or welfare associations sorted in alphabetical order by the name of the association. A: alpaca; L: llama.

Source	Association	Source	Country	Year	Species	Scale	Description
A1	Alpaca Association New Zealand	(Alpaca Association New Zealand 2012)	New Zealand	2012	A	1-5	The BCS is described in a graphical information sheet "Paddock Card-Body Scoring". The examination is performed as a hands-on method, in which the backbone, the thorax behind the elbow and the chest are palpated. In addition, the area between the front and rear legs is visually examined. A detailed description is provided for each score. Schematic illustrations of the spine for each score are given. The examiners are advised to check the BCS regularly and be aware of sudden changes in the BCS. The ideal score for adult huacaya alpacas is given as 3, for adolescent huacaya alpacas under one year as 4 and as 5 for suri and crias <6 months.
A2	Australian Alpaca Association (“Alpaca fact sheet #4”)	(Australian Alpaca Association 2008)	Australia	2008	A	1-5	The description of the BCS takes place in an information brochure "Body condition Score (BCS) of Alpacas". The scoring is considered more accurate than the weighing of the animals. The examination should be done as a hands-on method, as the visual examination could lead to misinterpretations. The area over the central backbone near the last ribs as well as the ribs at the point of the elbow are palpated. The hairless area between the front legs are examined by palpation or observation. Schematic drawings with cross-sections of the spine, as well as the body sites for examination, are also provided. It is recommended not to include the pelvis in the examination. The BCS should be practised, whenever the animals are handled. The ideal score is given as 2.5-3.5 for wethers, non-pregnant mature females,

							mature males and working males and 3-3.5 in pregnant females and growing animals (<15 months).
A3	Australian Alpaca Association (“Alpaca Advice”)	(Australian Alpaca Association n.g.)	Australia	n.g.	A	1-5	The description of the BCS can be found in the information brochure “Alpaca Advice – Body Condition Scoring”. The scoring is considered more accurate than the weighing of the animals, it is figured out, that there is no substitute for hands-on. The advantages of assessing a BCS compared to weighing are highlighted. The area over the central backbone near the last ribs as well as the ribs at the point of the elbow are palpated. Schematic drawings with cross-sections of the spine, as well as the body sites for examination (but other illustration than in (A2)), are also provided. It is recommended not to include the pelvis in the examination. The BCS should be practised, whenever the animals are handled. The ideal score is given as 2.5-3.5 for wethers, non-pregnant mature females, mature males and working males and 3-3.5 in pregnant females and growing animals (<15 months).
A4	British Alpaca Society (Turner)	(Turner 2014)	UK	2014	A;L	1-5	The description of the BCS can be found in the “Welfare Guide” of the British Alpaca Society. The examination is described as a hands-on method, as the visual examination is difficult due to the hair coat. A palpatory examination of ribs, spine, pelvis and rump is recommended. The areas between the front and hind legs are additionally examined visually. For each score (1 to 5) a detailed description of the findings is also given. Similar schematic drawings like in (A1-3) are provided. The ideal score is given as 3 for animals over two years of age, 4 to 5 for suri-type camelids, 4 for huacayas under 1 year of age, and 5 for crias <6 months of age.

A5	British Alpaca Society (“Alpaca fact sheet #4”)	(British Alpaca Society 2018)	UK	2018	A	1-5	The BCS is described in the information brochure "Alpaca Body Condition Scoring (BCS)". The description is based on the description of (A2). The scoring is considered more accurate than the weighing of the animals. the examination should be done as a hands-on method, as the visual examination could lead to misinterpretations. The area over the central backbone near the last ribs as well as the ribs at the point of the elbow are palpated. The hairless area between the front legs are examined by palpation or observation. Schematic drawings with cross-sections of the spine, as well as the body sites for examination, are also provided. It is recommended not to include the pelvis in the examination. The BCS should be practised, whenever the animals are handled. The ideal score is given as 2.5-3.5 for wethers, non-pregnant mature females, mature males and working males and 3-3.5 in pregnant females and growing animals (<15 months).
A6	Camelid Community - Standards of Care Working Group	(Camelid Community - Standards of Care Working Group 2005)	USA	2005	A;L	1-10	The BCS is described in an information brochure on "Recommended Practices in Caring For Llamas & Alpacas". Regular weighing and body scoring should take place equally. The examination should be done as a hands-on method, as fiberecan distort the body contour by palpating the backbone in mid-back as well as the ribs behind the forlegs. Schematic illustrations showing the back from behind at 3 different scores are provided. The examination should be performed at least twice a year. The optimal BCS is given as 5 to 6.
A7	New Zealand Government	(New Zealand Government 2018)	New Zealand	2018	A;L	1-5	The BCS is described in the New Zealand Government's “Code of Welfare: Llamas and Alpacas”. Checking the nutritional status should be done by regular weighing. Especially for crias, body weight is seen as a better parameter than BCS. The examination is described as a hands-on method and takes place by palpation of

							the fat and muscle covering over the ribs, spine, rump, chest and pelvis. In addition, the area between the front and rear legs is visually examined. A detailed description is provided for each score, similar illustrations as in (A1-3) are provided. The ideal score is given as 3.

Table S4: Detailed overview on sources about body condition scores for llamas and alpacas from veterinary services sorted in alphabetical order by the name of the veterinary service. A: alpaca; L: llama.

Source	Association (Author(s))	source	Country	Year	Species	Scale	Description
V1	Camelid Veterinary Services (Whitehead)	(Whitehead 2019)	UK	2019	A	1-10	The description of the BCS can be found in the information brochure "Body Condition Scoring Alpacas". On the homepage, where this file can be downloaded, there is also a youtube video, which conveys the contents as an instructional video. In addition, schematic illustrations of the body sites to be examined and a cross-section of the spine at 5 different scores are given. The practical examination is divided into two main and two secondary sites. The main sites consist of palpation of the mid-back as well as the last ribs of the ribcage. In fat animals, the areas between the front and hind legs should also be visually assessed. the examination of the BCS should be performed monthly and the color of the mucous membranes should be recorded at the same time. Weighing of the animals should be done especially in crias and for correct dosage of medication. The ideal BCS is given as 5.5 to 6, but differences in breed, sex and age should be considered.
V2	Criagenesis (Vaughan)	(Vaughan 2019)	Australia	2019	A	1-5	The BCS is described in the informational brochure: "Tips for drought management in alpaca herds". The author refers to (A2) and uses the same schematic figure for illustration. The examination should be done by palpation of the musculature over the backbone at the last ribs. Assessment of the BCS should be done regularly and the ideal BCS is given as 2.5 to 3.
V3	Criagenesis (Vaughan)	(Vaughan 2015)	Australia	2015	A	1-5	The BCS is described in the information brochure "Top ten tips of alpaca nutrition". The author refers to (A2) and uses the same schematic figure for illustration. When examining the BCS, backbone near the last ribs should be palpated first and then the ribs at the point

							of the elbow. Palpatory assessment of the pelvis should not be performed. Finally, the areas between the forelegs and hindlegs should be examined both visually and by palpation. The author emphasizes that practise and consistency are important for the assessment of a BCS. The ideal BCS is given as 2.5 to 3 for maintenance; working males, non lactating and non pregnant females and 3 for growth and females at full term.
V4	Criagenesis (Vaughan)	(Vaughan n.g.)	Australia	n.g.	A	1-5	The BCS is described in the information brochure "Feeding alpacas to maximise their reproductive potential". The author refers to (A2). A schematic figure showing the cross section of the spine is included for illustration. When examining the BCS, backbone near the last ribs should be palpated first and then the ribs at the point of the elbow. A palpatory assessment of the pelvis should not be performed. Finally, the areas between the forelegs and hindlegs should be examined both visually and by palpation. The author emphasizes that practise and consistency are important for the assessment of a BCS, several advantages of BCS over weighing are mentioned. The ideal BCS should be 2.5 in nonworking males and wethers; non-lactating and non-pregnant females. The BCS of the dam at birth should be 3. During lactation, the BCS should decrease to a minimum of 2.25. Working males and growing animals should have a BCS of 2.5 to 3.
V5	Endell Veterinary Group (Walton and Smith)	(Walton and Smith 2020)	UK	2020	A	1-5	The description of the BCS can be found in an information brochure about "The Healthy Alpaca". The examination is performed by palpation of the central backbone near the last ribs. The description of the BCS is supplemented by schematic illustrations of the body site, as well as a cross-section through the spine. The ideal BCS is given as 3, but influences by sex, age and pregnancy are mentioned.

V6	National Animal Disease Information Service<br>(Potter)	(Potter 2012)	UK	2012	A	1-5	The BCS is described in an information brochure on "The healthy alpaca". The examination is described as a hands-on method by palpation. it is pointed out that the visual examination can lead to misinterpretations due to the thick fleece. During the examination, the fat deposits of the central backbone in the area of the last rib, as well as the thorax in the area of the elbow are palpated. A detailed description is available for each score with schematic illustrations of the examined body sites, and cross sections of the spine. It should be noted that the pelvis should not be included in the examination, as even obese alpacas feel bony there. The examination should be performed regularly. The ideal score is given as 2.5-3.5 for wethers, non-pregnant mature females, mature males and working males and 3-3.5 in pregnant females and growing animals (<15 months).
V7	Pennsylvania State University<br>(Van Saun)	(Van Saun 2013)	USA	2013	A;L	1-5	The description of the BCS can be found on the homepage of the College of Agricultural Sciences at Pennsylvania State University. The author illustrates the survey of the BCS on a detailed picture board, which can also be found in other sources (T7-9; S8). The loin behind the ribs and in front of the pelvis should be palpated. Furthermore, the paralumbar fossa should be included in the examination, as well as the area between the forelegs and hindlegs should be assessed visually. In contrast to the other descriptions of van Saun no statement is made about palpation of the pelvis. The examination of the BCS should be done periodically, early to mid pregnancy and early to mid lactation are indicated as important times. In addition, a tabular overview of how to respond to changes in BCS in different groups of animals is provided. The ideal BCS is given as 2.5-3.5 in growth and for maintenance; 3.0-3.5 in late pregnancy; and 2.5-3.0 in lactation.

References

Alpaca Association New Zealand (2012) Paddock Card – Body Scoring. https://www.alpaca.org.nz/index.php/download_file/view/161/114/. Accessed 20 March 2020

Australian Alpaca Association (2008) ALPACA FACT SHEET #4 - BODY CONDITION SCORE (BCS) OF ALPACAS. https://alpacalibrary.com/media/blogs/husbandry-for-beginners/quick-uploads/p177/alpaca_fact_sheet_4_body_condition_sep_2013.pdf?mtime=1525215224. Accessed 17 October 2023

Australian Alpaca Association (n.g.) Alpaca Advice - Body Condition Scoring. https://alpaca.asn.au/wp-content/uploads/2020/07/AAA-Advice-Body-Condition-Score-.pdf. Accessed 17 October 2023

Bennett MM, Richards NL (2015) Camelid wellness. Vet Clin North Am Exot Anim Pract 18 (2):255-280. https://doi:10.1016/j.cvex.2015.01.006

British Alpaca Society (2018) ALPACA FACT SHEET#4. https://www.bas-uk.com/wp-content/uploads/2018/11/Fact-Sheet-4-Body-Condition-Scoring.pdf. Accessed 17 October 2023

Bromage G (2006) Feeding and Nutrition. In: Bromage G (ed) Llamas and Alpacas: A Guide to Management. The Crowood Press Ltd, Ramsbury, Marlborough, pp 34–45

Camelid Community Standards of Care Working Group (2005) Recommended Practices in Caring For Llamas & Alpacas. https://d2sc3az8tudm6u.cloudfront.net/73c3-256226-recommended-practices.pdf?versionId=7nxRTsivb9Yi8tO3PgTaFiUr_O.A_TGB. Accessed 17 October 2023

Duncanson GR (2012) Nutrition and Metabolic Diseases. In: Duncanson GR (ed) Veterinary Treatment of Llamas and Alpacas. CABI, Oxfordshire, UK, pp 13–21

Edmonson A, Lean I, Weaver L, Farver T, Webster G (1989) A body condition scoring chart for Holstein dairy cows. J Dairy Sci 72 (1):68-78. https://doi.org/10.3168/jds.S0022-0302(89)79081-0

Folkesson P (2007) Alpacka - en utfodringsstudie i fält. A field study on feeding of Alpacas in Sweden. Thesis, Swedish University of Agricultural Sciences Uppsala

Fowler ME (1998) Feeding and Nutrition. In: Fowler M (ed) Medicine and Surgery of South American Camelids. llama, alpaca, vicuña, guanaco, 2nd edn. Iowa State Univ. Pr., Ames, Iowa, pp 12-48

Fowler ME (2010) Feeding and Nutrition. In: Fowler ME (ed) Medicine and Surgery of Camelids, 3rd edn. Blackwell Publishing, Ames, Iowa, pp 17-58. https://doi.org/10.1002/9781118785706.ch2

Garnsworthy PC (2006) Body condition score in dairy cows: targets for production and fertility. In P.C. Garnsworthy PC, Wiseman J (eds.), Recent Advances in Animal Nutrition, Nottingham University Press, Nottingham, UK (2006), pp 61-86. https://doi:10.5661/recadv-06-61

Gomez F (2011) Nutritional Assessment, Nutritional Requirements and Forage Analysis of Llamas and Alpacas. Faculty Publications and Other Works - Large Animal Clinical Sciences. https://trace.tennessee.edu/utk_largpubs/24. Accessed 20 September 2023

Hilton C, Pugh D, Wright J, Waldridge B, Simpkins S, Heath A (1998) How to determine and when to use body weight estimates and condition scores in llamas. Veterinary medicine 93 (11):1015-1018

Johnson LW (1994) Update. Llama nutrition. Vet Clin North Am Food Anim Pract 10 (2):187-201. https://doi:10.1016/s0749-0720(15)30554-5

Johnson LW (2014) Physical examination and conformation. In: Cebra C, Anderson D, Tibary A, Van Saun R, Johnson LR (eds) Llama and Alpaca Care: Medicine, Surgery, Reproduction, Nutrition, and Herd Health. Elsevier Saunders, St. Louis, MO, pp 328–337. https://doi.org/10.1016/B978-1-4377-2352-6.00030-4

Jones M, Boileau M (2009) Camelid herd health. Vet Clin North Am Food Anim Pract 25 (2):239-263. https://doi:10.1016/j.cvfa.2009.02.006

Lopez B (2021) Approach to veterinary management of adult camelids. In Pract 43 (6):329-337. https://doi:10.1002/inpr.81

McConnell T, Hoffman E (2006): On-Farm Assessment and Diagnosis. In: Hoffman E (ed) The complete Alpaca book, 2nd edn. Bonny Doon Press, Santa Cruz, California, pp 415–426

Morin D, Rowan L, Hurley W, Braselton W (1995) Composition of milk from llamas in the United States. J Dairy Sci 78 (8):1713-1720. https://doi:10.3168/jds.S0022-0302(95)76796-0

New Zealand Government (2018) Code of Welfare: Llamas and Alpacas. https://www.mpi.govt.nz/dmsdocument/46039-Code-of-Welfare-Layer-llamas-and-alpacas. Accessed 17 October 2023

Niehaus AJ (2022) Physical Exam and Diagnostics. In: Niehaus AJ (ed) Medicine and surgery of camelids. 4th edn. Wiley Blackwell, Iowa, pp 108-136. https://doi.org/10.1002/9781119583295.ch4

Potter T (2012) The Healthy Alpaca. National Animal Disease Information Service. https://www.nadis.org.uk/disease-a-z/camelids/the-healthy-alpaca/. Accessed 17 October 2023

Turner A (2014) Alpacas, llamas & guanaco - welfare guide 2014. British Alpaca Society. https://www.bas-uk.com/wp-content/uploads/2018/10/Welfare-Guide-ALPACAS-and-LLAMAS-2015.pdf. Accessed 17 October 2023

Van Saun R (2006) Feeding the Alpaca. In: Hoffman E (ed.), The complete Alpaca book. 2nd edn. Bonny Doon Press, Santa Cruz, California, pp 179–232

Van Saun R (2013) Body Condition Scoring of Llamas and Alpacas. PennState Extension. https://extension.psu.edu/body-condition-scoring-of-llamas-and-alpacas. Accessed 30 August 2022

Van Saun R (2022) Feeding and Nutrition. In: Niehaus AJ (ed) Medicine and Surgery of Camelids, 4th edn. John Wiley & Sons, Iowa, pp 55-107. https://doi.org/10.1002/9781119583295.ch3

Van Saun R (n.g.) Nutritional Overview: Forage, Feed, Minerals and Other Supplements. https://zywieniealpak.urk.edu.pl/zasoby/227/Nutrition_Overview_Van_Saun_2020.pdf. Accessed 17 October 2023

Van Saun RJ (2009) Nutritional requirements and assessing nutritional status in camelids. Vet Clin North Am Food Anim Pract 25 (2):265-279. https://doi:10.1016/j.cvfa.2009.03.003

Van Saun RJ, Herdt T (2014) Nutritional assessment. In: Cebra C, Anderson D, Tibary A, Van Saun R, Johnson LR (eds) Llama and Alpaca Care: Medicine, Surgery, Reproduction, Nutrition, and Herd Health. Elsevier Saunders, St. Louis, MO, pp 100-123. https://doi.org/10.1016/B978-1-4377-2352-6.00012-2

Vaughan J (2015) Top ten tips of alpaca nutrition. Criagenesis. https://criagenesis.cc/wp-content/uploads/2015/11/CriaGenesis-adult-nutrition-v2.pdf. Accessed 17 October 2023

Vaughan J (2019) Tips for drought management in alpaca herds. Criagenesis. https://alpaca.asn.au/new-south-wales/wp-content/uploads/sites/4/2019/09/Drought-feeding-and-planning-for-alpacas-information-written-by-Dr-Jane-Vaughan.pdf. Accessed 17 October 2023

Vaughan J (n.g.) Feeding alpacas to maximise their reproductive potential. Criagenesis. http://yaringaalpacas.com.au/files/Feeding%20to%20maximise%20reproductive%20potention.pdf. Accessed 17 October 2023

Wagener MG, Ganter M (2020) Body Condition Scoring in South American camelids. Prakt Tierarzt 101:684-696. https://doi.org/10.2376/0032-681X-2020

Walker PG (2018) Camelid 101 - what you always wanted to know but were afraid to ask! American Association of Bovine Practitioners Conference Proceedings 51 (2):194-199. https://doi.org/10.21423/aabppro20183143

Walton L, Smith H (2020) The Healthy Alpaca. Endell Veterinary Group. https://www.endellfarmvets.co.uk/images/EVG-Blog-03july-2020.pdf. Accessed 17 October 2023

Whitehead C (2019) Body Scoring Alpacas. Camelid Veterinary Services. https://www.ukalpacavet.com/wp-content/uploads/2019/05/How-To-BCS-Alpacas-May2019.pdf. Accessed 17 October 2023

Wiedner E (2022) Management of Llamas and Alpacas. Merck Veterinary Manual. https://www.merckvetmanual.com/exotic-and-laboratory-animals/llamas-and-alpacas/management-of-llamas-and-alpacas. Accessed 17 October 2023
